# Circulating HPV DNA in the Management of Oropharyngeal and Cervical Cancers: Current Knowledge and Future Perspectives

**DOI:** 10.3390/jcm10071525

**Published:** 2021-04-06

**Authors:** Eriseld Krasniqi, Maddalena Barba, Aldo Venuti, Laura Pizzuti, Federico Cappuzzo, Lorenza Landi, Silvia Carpano, Paolo Marchetti, Alice Villa, Enrico Vizza, Greta Giuliano, Marco Mazzotta, Daniele Marinelli, Sandra Gnignera, Cristina Vincenzoni, Vincenzo Stranges, Domenico Sergi, Antonio Giordano, Federica Tomao, Marcello Maugeri-Saccà, Giuseppe Sanguineti, Francesca Sofia Di Lisa, Silverio Tomao, Gennaro Ciliberto, Patrizia Vici

**Affiliations:** 1 Division of Medical Oncology 2, IRCCS Regina Elena National Cancer Institute, 00144 Rome, Italy; krasniqier@gmail.com (E.K.); pizzuti8@hotmail.com (L.P.); federico.cappuzzo@ifo.gov.it (F.C.); landi.lorenza@gmail.com (L.L.); silvia.carpano@ifo.gov.it (S.C.); gregiuliano@gmail.com (G.G.); marcomazzotta88@gmail.com (M.M.); daniele.marinelli@uniroma1.it (D.M.); gnignera.sandra@gmail.com (S.G.); domenico.sergi@ifo.gov.it (D.S.); maugeri.marcello@gmail.com (M.M.-S.); patrizia.vici@ifo.gov.it (P.V.); 2 HPV-UNIT-UOSD Tumor Immunology and Immunotherapy, IRCCS Regina Elena National Cancer Institute, 00144 Rome, Italy; aldo.venuti@ifo.gov.it; 3 Department of Medical Oncology, University Sapienza, 00185 Rome, Italy; paolo.marchetti@hotmail.it; 4 Endocrinology Unit, Fondazione Policlinico Universitario A. Gemelli IRCCS, Università Cattolica del Sacro Cuore, 00168 Rome, Italy; aliceally95@hotmail.it; 5 Gynecologic Oncology Unit, Department of Experimental Clinical Oncology, IRCCS-Regina Elena National Cancer Institute, 00144 Rome, Italy; enrico.vizza@ifo.gov.it (E.V.); cristina.vincenzioni@ifo.gov.it (C.V.); 6 Sapienza University of Rome, 00185 Rome, Italy; stranges.1730316@studenti.uniroma1.it; 7 Molecular Medicine and Center of Biotechnology and Sbarro Institute for Cancer Research, College of Science and Technology, Temple University, Philadelphia, PA 19122, USA; president@shro.org; 8 Department of Medical Biotechnologies, University of Siena, 53100 Siena, Italy; 9 Department of Gynecologic Oncology, European Institute of Oncology, IRCCS, 20141 Milan, Italy; federica.tomao@ieo.it; 10 Radiotherapy Department, IRCCS Regina Elena National Cancer Institute, 00144 Rome, Italy; giuseppe.sanguineti@ifo.gov.it; 11 Department of Radiological, Oncological and Anatomo-Pathological Sciences, Umberto I University Hospital, University Sapienza, 00185 Rome, Italy; francescasofia.dilisa@uniroma1.it (F.S.D.L.); silverio.tomao@uniroma1.it (S.T.); 12 Scientific Direction, IRCCS Regina Elena National Cancer Institute, 00144 Rome, Italy; gennaro.ciliberto@ifo.gov.it

**Keywords:** circulating HPV DNA, liquid biopsy, oropharyngeal cancer, cervical cancer

## Abstract

Human papillomaviruses (HPVs) are associated with invasive malignancies, including almost 100% of cervical cancers (CECs), and 35–70% of oropharyngeal cancers (OPCs). HPV infection leads to clinical implications in related tumors by determining better prognosis and predicting treatment response, especially in OPC. Currently, specific and minimally invasive tests allow for detecting HPV-related cancer at an early phase, informing more appropriately therapeutical decisions, and allowing for timely disease monitoring. A blood-based biomarker detectable in liquid biopsy represents an ideal candidate, and the use of circulating HPV DNA (ct-DNA) itself could offer the highest specificity for such a scope. Circulating HPV DNA is detectable in the greatest part of patients affected by HPV-related cancers, and studies have demonstrated its potential usefulness for CEC and OPC clinical management. Unfortunately, when using conventional polymerase chain reaction (PCR), the detection rate of serum HPV DNA is low. Innovative techniques such as droplet-based digital PCR and next generation sequencing are becoming increasingly available for the purpose of boosting HPV ct-DNA detection rate. We herein review and critically discuss the most recent and representative literature, concerning the role of HPV ctDNA in OPC and CEC in the light of new technologies that could improve the potential of this biomarker in fulfilling many of the unmet needs in the clinical management of OPC and CEC patients.

## 1. Introduction

Human papillomaviruses (HPVs) are a family of small epitheliotrophic double-stranded DNA oncoviruses. Consistent evidence supports their role in invasive malignancies, particularly at the level of genital mucosa, upper respiratory tract and skin [1]. Among the 170 types of HPV thus far identified [2], at least 12 of them are classified as high-risk (HR) for cancer of cervix, anus, vagina, vulva, penis and oropharynx (HPV types 16, 18, 31, 33, 35, 39, 45, 51, 52, 56, 58 and 59) [3]. HPVs are mainly spread by sexual contact and a persistent infection is associated with carcinogenetic mechanisms [4]. HPVs replicate inside the infected proliferating cells and have its genes transcripted, leading to the synthesis of six early proteins (E1, E2, E4, E5, E6 and E7) and then two late capsid proteins [1]. HPV explicates its carcinogenic effect not only by integrating into the host DNA, but also using alternative pathways characterized by episomal E2, E4 and E5 (E2/E4/E5) expression [5]. However, the expression of HPV E6 and E7 oncogenes represents the main carcinogenic mechanism of the virus, as these two proteins inhibit the tumor suppressor proteins p53 and pRb, respectively [6]. HPV infection accounts for 3% of cancers diagnosed in women and 2% of cancers diagnosed in men [7], mostly represented by cervical cancer (CEC) in woman, and oropharyngeal cancer (OPC) in men. Human papillomaviruses are involved in almost 100% of CECs and in approximately 35–70% of OPCs, depending on the geographical region [8]. Cervical cancer is related to the HPV16 and HPV18 subtypes in around 70% of cases [9], and its histology is represented by squamous cell carcinomas in about 80% of cases, while the 20% manifest as adenocarcinomas [10]. On the other hand, among the HPV-positive OPCs, about 95% are induced by HPV16, and more than 95% present with a squamous histology [3,11,12]. The incidence of HPV-positive OPC is increasing, while the HPV-negative counterpart has been declining [13]. In interconnection with HPV etiology, the most relevant risk factor for CEC and OPC is sexual behavior such as early beginning of sexual activity and elevated number of oral sex partners, which favor the spread of the infection [14,15]. Other impacting risk factors are represented by estrogen–progesterone oral contraception, high parity and other sexually transmitted infections for CEC [16], and tobacco and alcohol for OPC [17]. Being that the cervix is easily accessible anatomically, pelvic exams and Papanicolaou smears are extensively employed as screening tools for an early diagnosis of HPV-related lesions. However, effective screening strategies for HPV-related OPC are lacking [18]. Moreover, HPV infection by HR subtypes can be prevented by vaccine-induced immunization, thereby outplaying the possibility of the virus to cause cancer in women [19]. Epidemiological evidence suggests that prophylactic HPV vaccination significantly reduces the prevalence of oral HPV infection. Due to the slow uptake and long latency period, vaccination is expected to translate into a decreased incidence of HPV-related OPC within the next 40 years [20,21]. Another relevant aspect at a clinical level is that HPV infection has a prognostic and predictive value in CEC and especially OPC. In fact, patients with HPV-mediated OPC tend to be younger, show higher response rates to treatment and longer survival [22,23,24,25] with respect to patients with HPV-negative tumors. The role of HPV in OPC as a favorable prognostic factor is maintained also in relapsed and metastatic disease [26]. Overall, evidence shows that HPV-positive OPCs respond better to treatment when compared to HPV-positive CECs [27]. Concerning the impact of HPV on CEC clinical course, consistent evidence supports a prognostic value of the HPV genotype. For instance, HPV18-positivity in CEC has a negative prognostic value in the early stage [28] and is a biomarker of higher risk of death [29]. Moreover, patients with HPV18- and HPV58-positive CECs may derive more benefit from the addition of concomitant chemotherapy to radiotherapy compared to those with HPV16- and HPV33-positive tumors [30]. Different genomic variants of HPV16 have been shown to define CECs with different behaviors [31]. Furthermore, the differential expression of HPV genes in the host may have prognostic and/or predictive relevance. One of such genes is represented by E2, which is intact in only 39% of patients with HPV16-positive CEC. These patients show a trend towards better disease-free survival with respect to the counterpart with an E2 disrupted gene [32]. The diagnosis of HPV-related CEC and OPC is obtained by demonstrating the presence of HR HPV DNA in the tumor using either polymerase chain reaction (PCR) or in situ hybridization (ISH), and in the case of OPC by p16 immunochemistry, which is usually scored as positive if there is a strong and diffuse nuclear and cytoplasmic staining in more than 70% of malignant cells [22]. Regarding treatment, the therapy for localized CEC consists in primary radiation with external beam RT and/or brachytherapy when the surgery is not feasible [33]. For persistent, relapsed or metastatic CEC the first-line systemic treatment is based on platinum-combination chemotherapy [34]. On the other hand, OPC tends to present in a locally advanced stage, and generally requires a multimodal treatment, which can be delivered in most of cases using definitive cisplatin-based chemoradiation [35]. Treatment of recurrent or metastatic OPC is based on platinum-fluorouracil combination chemotherapy [36]. In recent years, immunotherapy with immune checkpoint inhibitors has improved clinical outcomes in the metastatic setting for both CEC and OPC [37,38].

In an era marked by the advent of personalized, precision-oriented and mini-invasive medicine, the utility of circulating HPV DNA should be appraised. In fact, being HPV the main pathogenic factor in cancers such as CEC and OPC that affect a relevant number of subjects, detecting and quantifying its DNA in blood, could help optimize several aspects of cancer management. The inherent fields of application span from early diagnosis and pretreatment assessment to de-escalation strategies, efficacy monitoring and post-treatment surveillance.

We herein review the most updated literature from the current scientific scenario concerning circulating HPV DNA in CEC and OPC. While doing so, we aim at clarifying two main interconnected aspects. First, we will address the main unmet needs in the clinical management of patients with these two diseases, with a specific focus on early diagnosis, monitoring of treatment and surveillance. Additionally, second, we will attempt to analyze inherent literature on the potential integrating role of plasma detected HPV DNA in fulfilling current gaps in these patients’ management.

## 2. Materials and Methods

### 2.1. Literature Search

An electronic search of the PubMed database was performed to obtain key literature on a topic of interest published between 1 January 2000 and 10 January 2021. The following search terms were combined: cervical cancer; carcinoma of the cervix; oropharyngeal cancer; head and neck squamous cellular cancer; early diagnosis; treatment; surveillance; human papilloma virus DNA; cell free tumor DNA; circulating HPV DNA; PCR; droplet digital PCR and next generation sequencing. Further details on the search strategy are reported in Appendix A.

### 2.2. Search Results and Literature Screening

**Figure 1:**
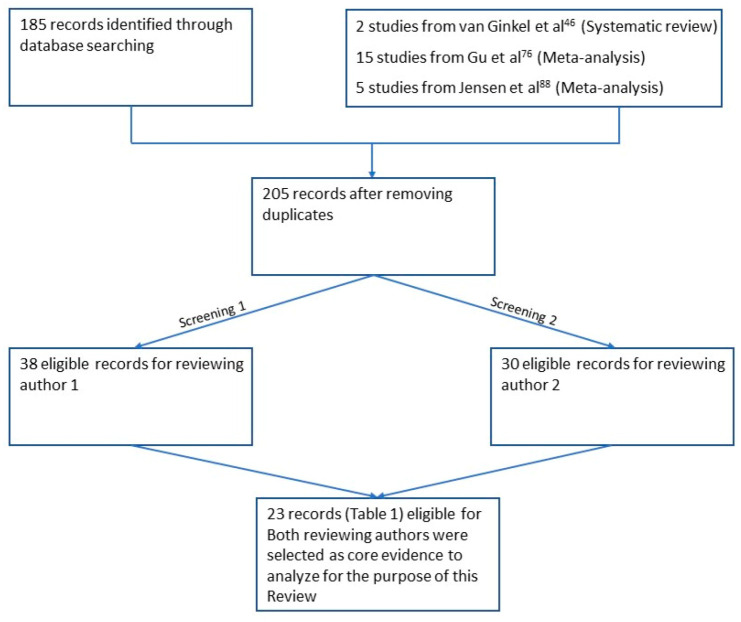
The flow diagram describes the strategy employed for literature research and screening.

The search performed according to the aforementioned criteria yielded 185 unique citations, which were included into a dedicated database (DB). This latter was independently screened by two reviewers. While the reviewers’ agreement was not required at the first screening round, based on the titles and abstracts of each publication initially selected, only those references for which both the reviewers agreed on inclusion following full text screening were finally judged suitable for inclusion in the current review and further considered. The flow diagram for the entire procedure is displayed in Figure 1.

## 3. Unmet Needs in HPV-Related CEC and OPC for Early Detection, Prognosis, Treatment Efficacy Prediction, Treatment Monitoring and Post-Treatment Surveillance

The pretreatment assessment, treatment monitoring and post-treatment surveillance for HPV-related cancers include physical examination, endoscopic evaluation and imaging studies. After definitive treatment of CEC and OPC, patients enter a follow-up program for surveillance. In both cases, imaging is generally performed at 3-months from the end of treatment and then repeated periodically. PET-CT imaging after radiotherapy in head and neck squamous cell cancer (HNSCC) has a high negative predictive value for residual disease at 3 months and has become the gold standard [39,40]. Local investigations, such as cervical/vaginal cytology for CEC and fiberoptic naso-pharyngo-laryngoscopy for OPC, are usually performed. Physical examination and patients’ reporting on recent events and disturbs integrate the previously cited assessment. However, while pretreatment endoscopies and radiologic investigations remain crucial for decision making at the time of treatment assignment, they become impractical during treatment, and even less feasible in course of post-treatment surveillance. Moreover, specific and minimally invasive tests are needed at the current time for detecting HPV-related cancer at an early phase. Indeed, since overall survival is strictly connected to the stage at diagnosis the same tests could be used also to better inform therapeutical decisions, facilitate disease monitoring and eventually allow for more conservative and efficacious treatments [41]. A blood tumor biomarker represents the favorite candidate for such scopes. Prior attempts to identify similar biomarkers have led to the squamous cell carcinoma antigen (TA-4), which has been investigated in CEC, demonstrating some degree of correlation with the disease stage [42] and relapse [43], but very low sensitivity in treatment monitoring [42]. This latter and other markers, such as the carcino embryonic antigen (CEA), tissue polypeptide antigen (TPA), tissue polypeptide specific antigen (TPS) and cytokeratin-19 fragments (CYFRA 21-1), have been long investigated as biomarkers of tumor activity and response to treatment in head and neck squamous cell cancer (HNSCC), including OPC, and have shown an acceptable sensitivity [44]. However, all these markers generally present low specificity for HPV-related cancer.

Liquid biopsy, a minimally invasive detection method for molecular biomarkers in body fluids, is a novel instrument with extraordinary potential in HPV-related cancers such as CEC and OPC. A systematic review inspected evidence concerning the current status of liquid biopsy in head and neck squamous cell carcinoma (HNSCC) patients by identifying and qualifying all published studies on the diagnostic or prognostic value of cell-free nucleic acids for post-treatment disease monitoring and/or disease outcome definition [45]. A relevant number of studies were identified, which investigated the prognostic and monitoring utility of biomarkers such as circulating miRNAs or ctDNA by targeting mutations, allelic imbalances, hypermethylation or HPV-DNA. Of these studies, two found a statistically significant association between nucleic acid levels (tumor DNA targeted by allelic imbalances and HPV-DNA) in blood and/or saliva and prognostic outcome.

Hence, exploiting the molecular correlates of the HPV infection in the context of HPV-associated cancer could offer an inherent high specificity, while sensitivity would be mostly related to the detection method that is used. HPV-infection induces the production of specific antibodies against virus-specific antigens. HPV16 E6 antibodies have high sensitivity and specificity in the case of OPC [46]. The inherent limit is represented by the fact that the antibody level remains elevated after definitive treatment, which precludes its use as a tumor marker for surveillance. The use of anti-HPV antibodies as tumor makers is even more limited for CEC, since patients with HPV infection could be antibody-positive even if they have not developed a cervical malignancy [47].

Moreover, in relation to the more favorable prognosis of HPV-positive OPC, finding efficacious deintensification strategies is critical [48]. From this perspective, tumor biomarkers that can allow for a more granular stratification and treatment optimization of HPV-associated OPC are strongly needed.

An effective tumor biomarker could be used to select patients for whom imaging tests beyond the 3-month post-treatment PET and/or RMN and local, cytology and endoscopy may be omitted, therefore improving the quality of life, without affecting survival outcomes [49,50]. On this basis, the use of the HR HPV DNA itself as a plasma biomarker in HPV-related tumors could represent an appropriate choice. A useful premise to the following paragraphs is represented by the need of applying standardized operative procedures (SOPs) to the preassessment handling of the biological samples analyzed throughout the different methods. Indeed, differences in the SOPs may significantly impact results from the biomarkers’ assessment and should be carefully considered when interpreting each study results.

## 4. Liquid Biopsy and Cell Free HPV DNA

Cell-free circulating nucleic acids in the form of DNA and RNA, of genomic, mitochondrial and viral origin, were first reported in the ‘40s [51]. Normal cells can release free nucleic acids into the circulation after apoptosis or even actively in the form of small DNA segments. When cancer develops, an amount of cell-free DNA (cf-DNA) is contributed by cancer cells and its specific fraction will depend on the total number of tumor cells, their proliferation rate and other pathological characteristics of the tumor [52]. Similarly, to normal cells, cancer cells release free DNA by various mechanisms, including apoptosis, necrosis or even by active secretion [53]. The tumoral fraction of the cf-DNA is called cell-free tumoral DNA (ct-DNA) and incorporates tumor specific somatic mutations, microsatellite alterations and epigenetic modifications [54].

The detection of ct-DNA in plasma is an innovative technique for obtaining tumor DNA from cancer patients, allowing a minimally invasive method to detect information about the cancer genome. Ct-DNA has the potential of being a highly specific tumor marker, even though sensitivity is low in that mutant ct-DNA fragments represent a small fraction of the total cf-DNA [55]. Recent progress in the detection and analysis of circulating tumor cells and ct-DNA have set the basis for the use of liquid biopsies for cancer diagnosis and monitoring. Ct-DNA could potentially be useful for screening purposes, prognosis definition, decision making and efficacy assessment [56,57]. Additional and potentially extremely appropriate uses of ct-DNA may relate informing decisions concerning the wide range of emerging targeted therapies, and monitoring relapse/recurrence [58]. The discovery of the epidermal growth factor receptor (EGFR) and Kirsten Rat Sarcoma virus (KRAS) mutations in cf-DNA, for example, has fuelled interest in lung and colorectal cancer management techniques, which have been shown to be useful for tracking treatment response and prediction of relapse [59,60]. A blood-based surveillance test for early detection of recurrence for breast, colorectal and bladder cancers using circulating DNA tumor assays has already been successfully validated [61,62,63]. One of the key drawbacks in the use of ct-DNA is the challenge in discriminating between tumor and host DNA. However, this limitation would not be a concern in HPV-related cancers, as any observed cf-DNA containing one or more copies of the HPV genome is derived by default from transformed cells.

As already described, HR HPV types integrate into the host genome and use the cellular instruments to express E6 and E7 for oncogenesis [64]. After integration, HPV genes behave in the same way as other genes of the human genome. HPV-related cancer cells and HPV ct-DNA may harbor genomic variations that can be released into the bloodstream during proliferation or apoptosis and that can be used as tumor markers. Ct-DNA in which HPV genome is integrated is detectable in the plasma of around 95.0% of patients affected by HPV-positive cancers, such as CEC and OPC [65,66,67]. The feasibility of the use of the cancer causative virus as a biomarker for a specific cancer has already been shown in the case of nasopharyngeal carcinoma (NPC), where plasma Epstein–Barr virus DNA can successfully detect subclinical tumors [68].

Moreover, the search for viral ct-DNA offers technical advantages over single nucleotide variant detection in liquid biopsy assays because of the size of the viral genome, its different nature from the human genome and because of the presence of multiple copies of the viral genome per tumor genome. All these factors may contribute to increase sensitivity of viral tumor DNA. HPV-induced cancers represent an optimal model to monitor ct-DNA by detecting the oncogenes E6 and/or E7 of HPV, which are a synonym of carcinogenic transformation. Moreover, nucleic acids incorporating HPV genome, particularly E6 and E7 DNA, have not been detected in the serum of patients with a simple HPV infection, but only in patients affected by HPV-related cancer [65].

In more detail, studies have demonstrated the potential usefulness of the ct-DNA use for CEC and OPC monitoring [69,70], and current techniques offer comparable predictive potential with personalized ct-DNA monitoring of other cancer types [60,61,62,63]. However, HPV ct-DNA monitoring offers the advantage of achieving equivalent accuracy without needing to develop patient-specific genomic assays.

## 5. Liquid Biopsy for HPV DNA Detection in CEC and OPC

Two decades ago, the first signals for the potential use of circulating HPV DNA (HPV ct-DNA) as a tumor marker in CEC began [67]. Since the beginning, the emphasis was put on the HPV ct-DNA’s potential to provide a good basis for CEC early diagnosis and prognosis information. The detection of HPV DNA in the serum of patients is a biomarker of poor CEC prognosis, as it correlates with lymphovascular invasion, nodal disease and larger tumors [66]. The largest part of patients with stage I CEC has detectable cf-DNA with integrated HPV DNA that reflects tumor burden [71]. Serum HPV DNA also correlates with relapse [72]. Serum HPV DNA negativity reached after radical CEC treatment tends to remain stable in patients with no tumor recurrence. Conversely, serum HPV DNA levels have shown increases in patients with CEC recurrence. Such increases on average anticipated approximately 2.5 months of the onset of clinically-instrumentally detectable disease recurrence [73]. Evidence has also shown that the level of serum HPV DNA may correlate with the burden of CEC disease, as it has been found to be higher in patients with metastatic disease compared to those with local recurrence [67]. In a study of patients with metastatic CEC, 100% of the HPV DNA was found in the serum and the sample was also used to classify the HPV genotype [74]. A meta-analysis examined the role of ct-DNA in CEC and found that detection of HPV ct-DNA in CEC patients could be used as a high-specificity and moderate-sensitivity, non-invasive early dynamic tumor biomarker [75].

The detection of ct-DNA using liquid biopsy has increasingly attracted the scientific community interest also for OPC [76]. Plasma circulating tumor HPV16 DNA (HPV16 ct-DNA) is detectable in the majority of patients with HPV16-associated OPC [77,78,79]. Indeed, approximately 95% of patients with HPV-related OPC have detectable serum HPV DNA at the time of diagnosis [77,80]. Elevated pretreatment levels of circulating HPV DNA correlate with higher nodal and overall stage in OPC [81,82]. Serum HPV DNA can be also be used to monitor for any residual disease after CRT [83]. Limited prior data has shown that HPV16 ct-DNA levels become largely undetectable post-CRT in most patients, and that HPV16 ct-DNA levels may increase at the time of disease recurrence [83,84]. Dynamic variations of serum HPV ct-DNA levels are associated with response to treatment of localized or metastatic HPV-related OPC [80,83,85]. Moreover, rising levels of circulating HPV DNA have been shown to anticipate the diagnosis of HNSCC recurrence by imaging exams [86]. A meta-analysis addressing circulating HPV DNA in patients with HNSCC showed that this tool is promising for the surveillance of recurrence [87]. For circulating HPV-DNA in post-treatment blood, they found a pooled sensitivity for recurrence of 54% (95% CI: 32–74%) and a pooled specificity of 98% (95% CI: 93–99.4%) with an area under the curve (AUC) of 0.93. Both the positive predictive value and the negative predictive value were high, i.e., 93% and 94%, respectively. These prior studies retrospectively analyzed limited subsets of patients using real-time PCR.

Unfortunately, when using conventional PCR, the detection rate of serum HPV DNA is about 12–45% in patients with CEC and ranges from 19% to 79% in patients with gross disease OPC [66,67]. Moreover, a standard cut-off for the detected HPV DNA copy number for the positivity of the test has not been defined.

## 6. New Methods to Increase Sensitivity of HPV ct-DNA Detection

The main techniques employed for the detection of ct-DNA in liquid biopsies include Sanger sequencing, pyrosequencing, digital PCR (dPCR) and next generation sequencing by tagged-amplicon deep sequencing (NGS). The simultaneous identification of alterations in multiple genes is feasible with the most innovative sequencing techniques. One of the simplest approaches used to increase the detection rate is the use of PCR primers that target shorter DNA fragments, since cf-DNA fragments have a length of approximately 180 bp [52]. Indeed, additional and more efficacious techniques are becoming increasingly available for the purpose of boosting the detection potential such as digital PCR (dPCR), droplet-based dPCR (ddPCR) and high throughput genome sequencing methods. The early diagnosis of tumors through the detection of ct-DNA could provide a significant decrease in morbidity and mortality of cancer patients. However, due in part to the low amount of tumor DNA released into the bloodstream and its dilution inside DNA derived from non-tumor cells, the adoption of ct-DNA for early cancer diagnosis is challenging. The introduction of new technologies such as ddPCR or optimized NGS could significantly improve the sensitivity, specificity and reliability of specific sequence detection [88]. Early attempts to use ddPCR for detecting HR HPV subtypes showed its high sensitivity (1.6 copies per sample for HPV16) and suggested that ddPCR could overcome the accuracy of classical quantitative PCR, which entails potential calibration bias [89]. A meta-analysis of 10 selected studies on the role of HPV ct-DNA as a reliable biomarker for CEC, showed at a descriptive level that the two studies that employed ddPCR as a detection method reported the highest sensitivity (83% and 90%) and specificity (100% in both), compared to the studies that adopted classic PCR [76]. Hence, ddPCR has surfaced as a newer method that facilitates ultrasensitive detection of even single copies of HPV DNA, holding a significant potential in quantification and monitoring of low-concentration targets [90].

### 6.1. Digital PCR

In a prospective study of 103 OPC patients, authors designed and validated a highly accurate ddPCR assay for absolute quantification of HPV ct-DNA relative to the subtypes -16, 18, 31, 33 and 35, representing the five most prevalent HR strains in this disease [81]. All tumors were p16-positive, although HPV status was known for only 52% of them. The ddPCR assay developed to amplify and quantify a specific region of the HPV16 E7 gene did not detect HPV16 ct-DNA in the plasma DNA extracted from 60 patients with HPV-negative neoplasm. The same assay revealed detectable levels of HPV16 ct-DNA in 84 out of 103 (82%) patients with HPV16-positive disease in the pretreatment phase. The remaining 19 samples, with undetectable HPV16 ct-DNA, were subsequently also analyzed by specific ddPCR assays for the alternative HR HPV strains (i.e., HPV-18, -31, -33 and -35), and 8 of them were positive for HPV DNA (1 HPV-31, 3 HPV-33 and 4 HPV-35). The final analysis concluded that HPV ct-DNA testing using this multianalyte ddPCR assay had 89% sensitivity and 97% specificity to identify HPV-related OPC [91] in patients with localized or locally advanced non metastatic newly diagnosed disease. In addition, there was some correlation between the disease stage and HPV16 DNA plasma levels. Specifically, a pattern of higher baseline plasma HPV16 DNA levels in patients with T2 tumors compared to T0/T1 tumors, and N2a/N2b with respect to N0/N1 extension of disease. However, lower levels of HPV ct-DNA were found in patients with T3/4 or N2c disease, conveying to the hypothesis that tumor burden might not be the only factor to explain the variation of pretreatment plasma levels of HPV ct-DNA in OPC patients. In fact, although HPV ct-DNA is detectable in most patients with newly diagnosed HPV-related OPC, low baseline HPV DNA might be also associated with the presence of a clinically higher-risk disease. Another important aspect that emerged from this study was based on the investigation of HPV ct-DNA clearance kinetics during CRT in a subset of 67 patients for whom weekly blood samples were collected and a post-treatment PET scan result was available. The weekly ct-HPV-16-DNA clearance kinetics was analyzed in the 40 patient subgroup who had >200 copies/mL baseline HPV ct-DNA. The HPV ct-DNA levels decreased in the course of CRT, and 80% of patients had completely eliminated HPV ct-DNA by the end of the treatment. In the further follow-up of these patients, HPV ct-DNA had cleared in 92%, 94% and 100% of patients by 6 months, 1 year and 2 years post-CRT, respectively. In an additional analysis, authors measured percent clearance of HPV ct-DNA at week 4 of CRT treatment, relative to pretreatment levels as a potential indicator of CRT sensitivity. Nineteen patients that showed a rapid HPV ct-DNA clearance of >95% at week 4, had a complete response to CRT.

The multianalyte ddPCR validated in this study [80] was used recently by the same research team to conduct another prospective biomarker clinical trial on patients with non-metastatic HPV-associated OPC [92], to assess the clinical utility of HPV ct-DNA for the surveillance of recurrence after definitive CRT treatment. With respect to recurrence, the study showed a 100% specificity, 99% sensitivity, 100% negative predicting value and 94% positive predicting value of the HPV ct-DNA detection by ddPCR.

Another study sought to determine the clinical utility of measuring serum HPV DNA using ddPCR in patients with HPV-related OPC [81], in different clinical settings. The ddPCR amplicon was built on the basis of an updated understanding of HPV16 and HPV33 subtype distribution and sequence variants. The experiments showed a 95.6% sensitivity and 100% specificity. A fraction of the analyzed patients had a low-burden disease, analogous to the desired subclinical volume, and in 100% of them HPV16 ct-DNA was detected. Overall, gross disease was only minimally related to HPV16 ct-DNA levels.

In another study [86], Hanna et al. employed a quantitative and ultrasensitive ddPCR optimized for the DNA detection in liquid biopsy of the 5 HR HPV subtypes associated with >99% of HPV-positive OPCs (HPV subtypes 16, 18, 31, 33 and 45). Twenty-two patients treated for advanced HPV-positive OPC were prospectively enrolled in this study. Results showed a clear correlation between tumor burden and ct-DNA viral load (R = 0.91, P = 2.3 × 10^−6^), and more distant anatomic locations generally predicted increasing median HPV ct-DNA viral levels. Moreover, plasma HPV DNA levels measured over several months in these patients declined significantly in those who obtained treatment response according to restaging scans. Furthermore, median plasma HPV DNA viral load was inversely correlated with overall survival (R = −0.48, P = 0.05), regardless of therapeutic intervention. This study adds further evidence on the potential of novel ddPCR technologies to revolutionize clinical management of HPV-related cancer.

In another retrospective study by Jeannot et al., authors utilized ddPCR to specifically investigate on the possible correlation between clinical stage and HPV ct-DNA levels at the time of diagnosis in 70 patients affected by HPV16- and HPV18-positive squamous cell carcinomas (47 cases with CEC, 8 cases with OPC and 15 cases with the anal canal tumor) [66]. Droplet dPCR was performed using HPV16 and HPV18 E7 gene specific primers. When using serum stored at both −20 °C and −80 °C, HPV ct-DNA was detected in 61/70 (87% sensitivity) and in none of 18 samples from women with HPV16-related high-grade cervical intraepithelial neoplasia used as a control (100% specificity). Specificity increased at 93% when using only serum stored at −80 °C (available for 27 patients only), with the detection of HPV ct-DNA also in two patients with microinvasive carcinomas. Concerning the main objective of the study, quantitative analysis showed that HPV ct-DNA levels in CEC patients were significantly higher when the clinical stage and tumor size increased (*p* < 0.01). ddPCR demonstrated to be a promising method for the detection and quantification of HPV ct-DNA also in subclinical stages showing its correlation with tumor dynamics.

A study evaluated pre-therapeutic HPV16 ct-DNA levels by ddPCR in patients affected by HPV16-positive OPC [93]. Pretreatment HPV16 ct-DNA was detected in 71% of patients, in which this baseline level positively correlated with T and N status and a positive trend for the M status. Most of the patients with negative baseline HPV16 ct-DNA had a stage I OPC, while all patients with metastatic disease had the biomarker detected. This study extended and completed three previous studies mentioned [65,81,85].

In a retrospective study Kang et al. [74] used duplex ddPCR assays for genotyping and quantifying HPV ct-DNA in the sera of 19 patients with HPV16- or HPV18-positive CEC. The results showed that HPV16 or HPV18 ct-DNA was specifically detected in all 19 HPV16- or HPV18-positive cervical cancer patients, with each identification matching that in the tumor tissues. None of the 45 healthy donors that were used as controls had detectable HPV DNA in their sera. Authors also evaluated the utility of HPV ct-DNA for HPV genotyping using multiple sequential serum samples from the patients with HPV-related CEC. Among all 87 samples that tested positive for HPV ct-DNA, 32 were HPV16-positive and 55 were HPV18-positive. There was a 100% match between the HPV types determined by HPV ct-DNA and those determined by tumor HPV DNA analyses for all samples. In this study authors also explored the feasibility of using HPV ct-DNA as a circulating biomarker in the follow-up of 9 CEC patients who had undergone an investigational tumor infiltrating lymphocyte (TIL) therapy. The levels of baseline HPV ct-DNA were not related to the treatment response. However, persistent HPV ct-DNA clearance was achieved in the two metastatic CEC patients with long-lasting complete responses.

The evidence provided by these studies suggests that HPV ct-DNA monitoring by ddPCR could represent a high-powered dynamic marker for HPV-associated cancer treatment assessment, especially in the context of new emerging antitumor therapies such as anticancer vaccines and immunotherapy.

The disadvantage of ddPCR is the necessity to use material for independent tests for each HPV subtype. If a single test had to be used for both CEC and OPC, then independent tests for HPV16, HPV18 and HPV33 are required. Another limit in relation to the potential of ddPCR for early disease screening, is the fact that HPV ct-DNA was detectable strictly in the cases with microinvasive CEC, while patients with high-grade intraepithelial lesions were negative for the biomarker [65].

An alternative to ddPCR is represented by next generation sequencing (NGS), which could capture multiple subtypes in only one test, and yield higher sensitivity.

### 6.2. Next Generation Sequencing

In a recent study, Han et al. hypothesized that a next generation sequencing (NGS) approach could outperform PCR-based methods for viral ct-DNA detection [93]. Hence, authors developed a novel NGS method for viral ct-DNA detection for both HPV and Epstein–Barr virus (EBV), which could carry out genome sequencing for both viruses by the hybrid capture technique. With regards to HPV in CEC patients, the study showed that the NGS method had a 10-times lower limit of the detection threshold when compared to digital PCR, with a sensitivity and specificity of 100%. The study showed that for virus-associated cancers, viral genome NGS provides dramatically greater sensitivity than PCR-based methods and enables viral subtyping and ct-DNA fragment length analysis.

In another prospective study [83], Lee et al. investigated the clinical utility of HPV ct-DNA in monitoring disease response following primary CRT in patients with locally advanced HNSCC, by developing and using an ultra-sensitive HPV DNA next generation sequencing (NGS) assay denoted as “HPV16-detect”. Separate test (55 patients) and validation (28 patients) cohorts were used for this purpose, and a total of 75 OPC patients were included (47 patients in the test cohort; 28 patients in the validation cohort). All patients were assessed for response by clinical examination and 18F-FDG PET-CT at 12 weeks after terminating CRT. Serial plasma samples were collected at baseline, 6 weeks and 12 weeks following the completion of CRT (at the time of PET-CT). The novel NGS assay (HPV16-detect) designed by the authors to detect HPV ct-DNA comprised a 39-amplicon single pool panel covering 34 distinct regions of the HPV16 genome, covering nucleotide variations in the most prevalent HPV16 subtypes and human reference genes (5 amplicons). NGS detection through HPV16-detect was validated in tumor tissue by comparing its efficacy to E7 mRNA detection using RT-PCR, considered as the gold-standard assay for this purpose. Results demonstrated 100% specificity and 100% sensitivity for HPV16-detect in determining HPV16 status in tumor tissue. Overall, 66% of patients in the test cohort and 61% of patients in the validation cohort had HPV-positive tumors (all of them being OPC). In the test cohort HPV16-detect assay demonstrated 100% sensitivity and 92.9% specificity in detecting HPV DNA in plasma compared to tissue. While, in the validation cohort it demonstrated 90% sensitivity and 100% specificity in assigning HPV status in plasma compared to p16 staining in tissue. HPV16-detect was also used to track plasma HPV16 DNA in patients with HPV16-positive tumors in order to identify residual disease. None of the 33 patients with HPV16-positive disease and complete PET-CT response had detectable plasma HPV16 DNA by HPV-detect at 12 weeks after CRT completion.

Lee et al. used a NGS assay also in another prospective study [94] to detect circulating HPV DNA in patients undergoing CRT for anal squamous cell carcinoma (ASCC), which is casually related to HPV in the majority of cases. In this case the authors developed and validated an ultrasensitive HPV DNA NGS assay, denoted as “panHPV-detect”, with the ability to comprehensively detect circulating DNA of eight different HR HPV genomes (16, 18, 31, 33, 35, 45, 52 and 58). Authors showed that the panHPV-detect NGS assay was 100% sensitive and 100% specific for the identification of ctHPV-DNA in plasma at diagnosis, and showed clinical utility by predicting response to CRT when measuring cHPV-DNA in the post-treatment period.

## 7. Discussion

Blood-based biomarkers represent an ideal potential modality for the early detection or surveillance of HPV-associated cancers such as OPC and CEC. In the recent years ct-DNA has gained much attention as a method of investigating and monitoring tumor biology and clinical status [84,95]. Ongoing research is appraising the potential role of circulating HPV DNA in HPV-related cancers for this purpose. In the tumor, HPV DNA is integrated into the host genome or is present in episomal form [96] and can be detected in the blood as part of the free tumor DNA. According to a recent meta-analysis, HPV16 E antibodies and circulating HPV DNA exhibited the strongest biomarker performance characteristics compared to other blood-based candidate biomarkers, such as microRNA expression profiles, cytokine levels, vitamins and cofactors and various gene polymorphisms [97]. The wide spectrum of inherent literature critically reviewed in this paper shows an increasing interest towards the potential role of HPV DNA detection in liquid biopsy for the management of OPC and CEC. Overall, the spectrum of potentials for the use of liquid biopsy in oncology practice include early detection, assessment of molecular heterogeneity of overall disease, monitoring of tumor dynamics, identification of genetic determinants for targeted therapy, evaluation of early treatment response and monitoring of minimal residual disease and assessment of resistance evolution in time. In the current era of major biotechnological innovation, these utilities are progressively obtainable by the detection of circulating HPV DNA in the plasma of patients affected by HPV-associated OPC and CEC, as an unambiguous biomarker of tumor DNA. In principle, there are no doubts on the clinical utility of circulating HPV DNA. However, the evidence that we analyzed clearly indicates that the degree at which it is possible to exploit such high potential provided by circulating HPV DNA, is directly dependent on the accuracy of the detection methods. In fact, the use of circulating HPV DNA was not clinically relevant as long as classical quantitative PCR was used for detection. With a high lower limit of detection threshold, and 60–70% sensitivity, this technique did not allow for a secure use of circulating HPV DNA in clinical practice as a biomarker of response to treatment or early relapse. However, with innovative PCR-based techniques such as ddPCR the real potential of circulating HPV DNA as an ideal biomarker in HPV-related OPC and CEC was unleashed. This method is able to reach over 90% sensitivity and specificity in the detection of HPV DNA in plasma, as the recent studies have demonstrated. Moreover, it has a high reproducibility inter- and intra-laboratories [98]. Digital dPCR assays can also be optimized to detect the DNA of more than one HR HPV subtype. When using this technique, circulating HPV DNA is able to inform on the HPV-positivity status of the tumor, provide information on the disease stage and burden, evaluate response to treatment by its plasma kinetics, predict relapse during surveillance, and offer the possibility for HPV genotyping.

With the recent advent of NGS as a cornerstone for tumor DNA sequencing, researchers have attempted to adapt the technique for the purpose of circulating HPV DNA detection. In this review we described the few accomplished studies to date that used NGS for HPV DNA detection in liquid biopsy. In 2013, NGS was successfully used for HPV genotyping [99], and in 2016 the identification of HPV infection in cancer tissue by targeted NGS was demonstrated to be feasible [100]. More recently, different assays have been validated for the detection of circulating HPV DNA, with amplicon-based or capture-based technologies and targeting one or more HR HPV subtypes. The first results are extremely promising, showing higher accuracy than digital PCR while offering a multitude of potential other advantages, such as higher coverage of HPV genome, the ability to detect more genotypes at the same time, shorter processing time, etc. Besides increasing the potential for the use of circulating HPV DNA in clinical practice, detection by NGS could unveil new possibilities for research. For instance, the site of HPV integration could have an impact on cancer progression (disruption of cancer suppressor genes, immunomodulatory genes, etc.), and different HPV variants have been shown to differ biologically and functionally, thereby affecting persistence and potentially the risk of cancer progression [101]. Recently, using a NGS technology called “Capture HPV” [102] on biopsies and circulating DNA material, five molecular signatures of HPV integration have been identified in HPV cervical cancer and correlated with survival, even though at a not statistically relevant extent. Investigations based on this new technology are actually in the process in HPV-related OPC. Moreover, specific in silico automated tools have been developed for multi-HPV type detection. These tools perform annotation and determination of site of HPV integration utilizing raw exome, transcriptome or whole-genome data as input with minimal requirement for third-party tools. The study from Mes and colleagues may possibly exemplify future perspectives and goals from one of the most flourishing research pipelines in this area. The authors investigated whether the addition of the detection of HPV DNA and specific copy number alterations (CNVs) in cf-DNA by NGS, could increase the sensitivity for the detection of ctDNA in HNSCCs, with respect to using only tumor-specific somatic alterations [103]. Plasma DNA underwent low coverage whole genome sequencing, to detect both CNAs and HPV DNA, and deep sequencing to detect mutations in 12 frequently altered cancer driver genes in HNSCC. Pretreatment plasmas of 40 patients and 20 non-cancer controls were used for analysis. Using the developed method, somatic mutations and CNAs were detected in plasma DNA of HNSCC patients in 67% and 52%, respectively. HPV-DNA in plasma was detected in 100% of patients with HPV-positive tumors, and not in plasma of patients with HPV-negative tumors or non-cancer controls. Combined analysis increased the detection rate of tumor DNA in plasma to 78%. This study demonstrates that the combined analysis of CNAs, HPV and somatic mutations in plasma of HNSCC patients is feasible and contributes to a higher sensitivity of the assay compared to single modality analyses. On this basis, the method could be furtherly optimized to detect HNSCC DNA also in HPV-negative patients.

Overall, the accuracy in the quantification of circulating HPV DNA in patients affected by OPC or CEC has dramatically improved in the last two decades in the transition from classical PCR techniques, to ddPCR and finally dedicated NGS (Table 1). Moreover, these new technologies have the potential to allow for the sensible detection of HPV DNA also in other body fluids, such as saliva [78].

The deep information uncovered with ddPCR and NGS technologies such as viral molecular status, genotype variants, integration of viral genes deletion and sites of integration could be extremely informative regarding the viral oncogenic process and could allow the possibility to furtherly stratify HPV-driven cancers, and optimize clinical management.

## Figures and Tables

**Table 1:** The main studies that investigated on the techniques and clinical utility of the circulating HPV DNA detection in patients affected by HPV-positive cervical cancer (CEC) and oropharyngeal cancer (OPC). Abbreviation: CEC = Cervical cancer; OPC = Oropharyngeal cancer; HNSCC = head and neck squamous cell carcinoma; SCAC = squamocellular anal carcinoma; NSCLC = non-small cell lung carcinoma; CRC = colorectal cancer; RT-PCR = Real Time polymerase chain reaction; ddPCR = droplet digital PCR; NGS = next generation sequencing; ctDNA = circulating tumor DNA.

Study	Tumor	Nr. of pts	DNA Extraction and Detection Technique	Main Study Outcomes
68. Human papillomavirus DNA in plasma of patients with cervical cancer. Pornthanakasem W et al. March 2001.	CEC	60	Classic PCR: Plasma was obatined by entrifuging blood specimens in EDTA anticoagulant at 3300 rpm for 10 min. Plasma were stored at −20 °C. Phenol/chloroform extraction method was used. Plasma DNA was purified on Qiagen columns. E performed. E6 primer was used for PCR.	The circulating DNA had low sensitivity. Only 6 out of 50 (12%) with HPV associated CC patients were demonstrated to have circulating HPV DNA.
74. Human papillomavirus DNA in sera of cervical cancer patients as tumor marker. Widschwendter A et al. December 2003.	CEC	94	Classic PCR: Serum was prepared and stored at −80 °C. Final experiments were conducted with the phenol/chloroform extraction method and the L1 primer was used for PCR EIA.	Serum samples taken at the date of diagnosis were HPV DNA positive in 45% of all investigated samples. HPV DNA was positive up to 423 days before the time of clinical diagnosis of recurrence in 10 out of 13 cases (median 72 days, range 0–423 days).
67. Clinical significance of serum human papillomavirus DNA in cervical carcinoma. Hsu KF et al. December 2003.	CEC	112	RT-PCR: Plasma was obtained by lowspeed centrifugation (1200× *g* rpm) for 5 min and stored at −80 °C. DNA was extracted with the High Pure Viral Nucleic Acid kit (Boehringer-Mannheim Biochemicals). L1 degenerate consensus primers and E7 type-specific primers was used for PCR.	The detection of circulating HPV DNA by RT-PCR showed a sensitivity of 45.2% and a specificity of 88.6%.
78. Quantitation of human papillomavirus DNA in plasma of oropharyngeal carcinoma patients. Cao H et al. March 2012.	OPC	64	RT-PCR: Plasma was obtained by centrifugation (3000× *g* rpm 4 °C for 10 min), and stored at −80 °C. DNA was extracted using the QIAamp Blood Mini Kit. L1, E6, and E7 primers were used for PCR.	The L1 primer had the highest detection rate of 68%, followed by qPCR for E6/7 (65%). When less sensitive primers (nested E6 and E7) and conventional PCR were used, plasma HPV DNA was positive in only 42% of patients.
72. Human papillomavirus mutational insertion: specific marker of circulating tumor DNA in cervical cancer patients. Campitelli M et al. August 2012.	CEC	16	RT-PCR: DNA was isolated from with QIAamp Min Elute Virus Spin Kit (Qiagen). RT-qPCR assay using Sybr^®^Green (Applied Biosystems) was designed to specifically amplify cell-virus junction DNA sequences or HPV16 E7 DNA.	ctDNA was detected in 11 out of 16 patients diagnosed with invasive cervical cancer. (Sensitivity 69%)
70. Optimization of circulating cell-free DNA recovery for KRAS mutation and HPV detection in plasma. Mazurek AM et al. 2013.	NSCLC, HNSCC, CRC	12 HNSCC (1 for HPV ct-DNA detection)	RT-PCR: Plasma was separated by double centrifugation at 300× *g* and 1000× *g*, both at 4 °C for 10 min and stored at −80 °C. Sherlock AX kit, Genomic Mini AX Body Fluids kit and QIAamp Circulating Nucleic Acid kit were compared for the yiled of DNA extraction. E6/E7 primers and probe were used for PCR.	In this study very low sensitivity of HPV16 detection in plasma by conventional PCR was highly improved by real-time probe-based HPV detection. Enlarging the input of plasma volume with a simultaneous decrease of elution volume improved HPV16 detection.
80. Saliva and plasma quantitative polymerase chain reaction-based detection and surveillance of human papillomavirus-related head and neck cancer. Ahn SM et al. September 2014.	OPC	93	RT-PCR: Blood samples were collected in citrate-containing blood tubes for plasma. Plasma samples were centrifuged at 1000× g rpm for 10 min, and the top layer was collected. DNA was extracted with the phenol/chloroform method. Primers and probes designed to amplify the E6 and E7 regions of HPV-16 were used for qPCR.	The sensitivity, specificity of combined saliva and plasma pretreatment HPV-16 DNA status for detecting tumor HPV-16 status were 76%, 100%. The sensitivities of pretreatment saliva or plasma alone were 52.8% and 67.3% respectively. The combined saliva and plasma post-treatment HPV-16 DNA status was 90.7% specific and 69.5% sensitive in predicting recurrence within 3 years.
79. Detection of somatic mutations and HPV in the saliva and plasma of patients with head and neck squamous cell carcinomas. Wang Y et al. June 2015.	HNSCC	47	PCR: Whole blood was collected. Four to 10 mL of plasma was used for DNA purification, with the average amount of plasma being 6 mL. DNA was purified from plasma using an QIAamp Circulating Nucleic Acid Kit. The presence of HPV16 and HPV18 was assessed using the primers specific for the E7 for PCR.	In plasma, tumor DNA was found in 80% of patients with oral cavity cancers, and in 86% to 100% of patients with cancers of the other sites.
85. Use of Human Papillomavirus 16 (HPV16) Cell Free DNA for Assessment of Response to Chemoradiation in HPV-Associated Oropharyngeal Cancer. Higginson D. S. et al. November 2015.	OPC	17	PCR: Eight to 10 mL of blood was collected in EDTA-lined vacutainer tubes. Samples were centrifuged at 820× *g* for 10 min. The upper plasma layer was further centrifuged at 16,000× *g* for 10 min to remove any remaining cell debris. Plasma was stored in microcentrifuge tubes at −80 °C for further analysis. DNA was extracted from the upper plasma layer (1 × 10^3^ mL) using a tool designed to isolate free-circulating DNA and RNA from human plasma or serum. Primers for quantitative PCR were selected via alignment of all known sequences of the HPV16 strain.	Of the 14 HPV-positive OPC plasma samples analyzed, 12 exhibited detectable HPV16 cfDNA with copy numbers ranging from 1 to 10,000 copies per mL of plasma.
83. Circulating human papillomavirus DNA as a marker for disease extent and recurrence among patients with oropharyngeal cancer. Dahlstrom KR et al. February 2016.	OPC	12	RT-PCR: A 30-mL blood sample was collected from each patient. Serum and plasma were isolated from the whole blood sample and stored at −80 °C prior to analysis. HPV16 DNA was extracted from a 500-μL volume using the QIAamp Blood Kit (Qiagen). HPV16 E6 and E7 regions were amplified by PCR.	Among the 218 patients treated at MDACC, 12 patients with HPV-positive tumors developed disease recurrence, and 7 of these 12 patients (58%) had HPV DNA detectable in pretreatment serum before treatment. For patients with HPV-negative tumors developed disease recurrence, none of whom had HPV DNA detectable in pretreatment serum.
102. Mechanistic signatures of HPV insertions in cervical carcinomas. Holmes A et al. March 2016.	CEC	72 (5 on circulating HPV DNA)	NGS: The ct-DNA was prepared from 200 μL of serum collected from a patient blood sample at the time of diagnosis, using the QIAamp viral DNA (QIAGEN) enrichment and blood kits. The probes were designed to detect the entire length of the HPV genomes from >200 HPV genotypes or variants. The library was then sequenced using Roche 454 Life Sciences Junior or paired-end reads using Illumina MiSeq.	Specific HPV genotypes were identified in 70/72 cases: HPV16 in 34 cases (49%), HPV18 in 18 cases (26%), HPV31 in four cases (6%), HPV45 and HPV73 in three cases each (4.3%), HPV33 and HPV68 in two cases each (2.8%) and one case each of HPV6, HPV42, HPV51 and HPV52 (1.4%). The same viral-junction patterns were identified in the blood and tumour samples from each patient.
66. Circulating human papillomavirus DNA detected using droplet digital PCR in the serum of patients diagnosed with early stage human papillomavirus-associated invasive carcinoma. Jeannot E et al. June 2016.	CEC, HNSCC, SCAC	47 CEC 8 HNSCC	ddPCR: Serum from patients with HPV16 or HPV16-associated invasive cancer were collected. Sera from the 8 patients with HNSCC, and from 35/47 patients with CEC were stored at −20 °C whereas others (27 cases) were stored at −80 °C. DNA was isolated in duplicate from 200 µL of serum or plasma, using the QIAamp Mini Elute Virus Spin Kit (Qiagen). Isolated DNAs were assayed with ddPCR and RT-qPCR. Primers and probes for the HPV16 E7 and HPV18 E7 were used.	In this study the detection of circulating HPV DNA showed a sensitivity of 87% and a specificity of 100%.
87. Circulating Cell-Free Human Papillomavirus DNA as a Marker of Treatment Outcome in Patients With HPV-Positive Squamous Cell Head and Neck Cancer After Radio(chemo) Therapy. Rutkowski T et al. October 2016.	HNSCC	477	RT-PCR: The cfHPV DNA status was assessed in plasma blood samples by TaqMan/PCR and confirmed in formalin-fixed paraffin-embedded tumor samples.	In this study, circulating HPV DNA was detected in 67 patients (14%). Circulating HPV DNA was subsequently assessed in 65 (13.5%) patients after treatment. Three patients (0.5%) presented uncured disease after the treatment and their cfHPV DNA remained detectable over the observations.
84. Predicting response to radical (chemo)radiotherapy with circulating HPV DNA in locally advanced head and neck squamous carcinoma. Lee JY et al. August 2017.	HNSCC	75	NGS: 20 mL of blood was centrifuged at 1500× *g* rpm. for 10 min and frozen at −80 °C. Prior to extraction, plasma samples were further centrifuged at 14,000× *g* rpm. for 10 min at 4 °C. DNA was extracted using the QIAamp Circulating Nucleic Acid Kit (Qiagen). For the validation cohort, DNA was extracted using the MagMAX Cell-free DNA Isolation kit (ThermoFisher Scientific). Plasma DNA was quantified using a Bio-Rad QX200 ddPCR system- using ribonuclease P. To detect HPV DNA, ‘HPV16-detect’, a novel NGS assay was designed using Ion Ampliseq Designer (ThermoFisher Scientific).	In pre-CCRT plasma, HPV-detect demonstrated 100% sensitivity and 93% specificity, and 90% sensitivity and 100% specificity for the test (27 HPV+) and validation (20 HPV+) cohorts, respectively.
75. Circulating Cell-free DNA for Metastatic Cervical Cancer Detection, Genotyping, and Monitoring. Kang Z et al. September 2017.	CEC	19	ddPCR: Blood samples were collected in red top vacutainer tubes (Becton Dickinson). After centrifuging at 2000× *g* for 10 min in a refrigerated centrifuge, serum samples were stored at –80 °C. DNA was extracted with an automated Maxwell RSC Instrument (Promega). To detect single copies of HPV16 or HPV18 DNA, a ddPCR method was developed using the sequences of the HPV16 or HPV18 E7 genes.	In blinded tests, HPV ccfDNA was detected in 19 of 19 (100%) patients with HPV-positive metastatic cervical cancer but not in any of the 45 healthy blood donors.
86. Plasma HPV cell-free DNA monitoring in advanced HPV-associated oropharyngeal cancer. Hanna GJ et al. September 2018.	OPC	17	ddPCR: Plasma was spun down from whole blood samples (1500 × *g* for 10 min (min)) and cell-free DNA (cfDNA) was extracted using the QIAamp Circulating Nucleic Acid Kit (Qiagen). E7 genes from the five dominant high-risk HPV subtypes (16, 18, 31, 33 and 45) were cloned into pUC57 plasmids (GenScript) as positive controls. ddPCR was performed.	Twenty-two patients with advanced HPV+ OPC were enrolled. Total tumor burden (TTB) strongly correlated with HPV cfDNA levels (R = 0.91, P = 2.3 × 10^−6^) at this cohort size. All participants demonstrated a corresponding change in their HPV cfDNA levels at a median of 16 days before restaging. Both TTB and median plasma HPV cfDNA levels negatively correlated with survival (R = −0.65, P = 0.01; R = −0.48, P = 0.05, respectively).
73. Liquid biopsy of HPV DNA in cervical cancer. Cheung TH et al. March 2019.	CEC	138	ddPCR: Plasma samples were collected at the time of study entry and stored at −70 °C prior to analysis, and later thawed and centrifuged at 2000× *g* at 4 °C for 10 min before DNA extraction. DNA was extracted using the QIAamp Circulating Nucleic Acid Kit Extraction (Qiagen). ddPCR was carried out using the ddPCR Supermix for Probes.	In this study HPV E7 and L1 sequences were detected in plasma cfDNA from 61.6% (85/138) of patients.
82. Detection of Early Human Papillomavirus-Associated Cancers by Liquid Biopsy. Damerla RR et al. April 2019.	OPC	97	ddPCR: Ten milliliters of whole blood were collected from each patient into cell-free DNA (cfDNA) BCT tubes (Streck) or BD Vacutainer K2 EDTA tubes (BD Biosciences). Plasma was separated first though centrifugation at 800× *g* for 10 min, followed by an additional centrifugation at 16,500× *g* for 10 min, and stored at −80 °C. DNA was extracted from 4 to 5 mL of plasma using Qiagen circulating nucleic acid kits (Qiagen). HPV16 and HPV33 ddPCR assays were designed using Primer3Plus.	The detection of circulating HPV DNA in this study showed a 95.6% sensitivity and 100% specificity.
81. Rapid Clearance Profile of Plasma Circulating Tumor HPV Type 16 DNA during Chemoradiotherapy Correlates with Disease Control in HPV-Associated Oropharyngeal Cancer. Chera BS et al. August 2019.	OPC	103	Multianalyte dPCR: Blood specimens were collected in 10 mL cell-free DNA BCT^®^ blood collection tubes (Streck 218962), and double-spun plasma (2000× *g*) was harvested within 3 days for storage at −80 °C. DNA was extracted using the QIAamp circulating nucleic acid kit (Qiagen). Primers and 5′ hydrolysis probes were designed to specifically detect a 75 bp amplicon within the E7 gene encoded by high-risk HPV strains 16, 18, 31, 33 and 35.	The detection of circulating HPV DNA in this study showed a sensitivity and specificity of respectively 89% and 97%.
94. Viral Genome Sequencing for Ultrasensitive Detection of Circulating Tumor DNA. Han K et al. September 2019.	CEC	18	NGS: Viral genome sequencing for HPV (HPVseq) was achieved by hybrid capture NGS.	The detection of circulating HPV DNA in this study showed a sensitivity and specificity of respectively 100% and 100%
104. Comprehensive multiparameter genetic analysis improves circulating tumor DNA detection in head and neck cancer patients. Mes SW et al. June 2020.	HNSCC	40	NGS: Samples of 4 × 6 mL whole blood were taken using EDTA vacutainers. Plasma was collected by centrifugation, and further purified with an additional centrifugation step at 20,162× *g* using a Hettich EBA 12 R microcentrifuge. was isolated using a Qiasymphony automated platform (Qiagen). Plasma NGS libraries were generated with a 5500 SOLiD™ Fragment Library kit and TruSeq adapters. lcWGS was performed on the plasma. An algorithm was developed to map reads from lcWGS to HPV genomes.	HPV DNA in plasma was detected in 100% of patients with HPV-positive tumors, and not in plasma of patients with HPV-negative tumors or non-cancer controls.
92. Plasma Circulating Tumor HPV DNA for the Surveillance of Cancer Recurrence in HPV-Associated Oropharyngeal Cancer. Chera BS et al. October 2020.	OPC	86	ddPCR: Blood samples were collected in 10-mL cell-free DNA BCT blood collection tubes (Streck). DNA was extracted using the QIAamp circulating nucleic acid kit (Qiagen). Validated digital PCR (dPCR) assays were performed on the QX-200 platform. E6 HPV16/E7 HPV18, 31, 33, 35 primers and probes were used.	The detection of circulating HPV DNA in this study showed a sensitivity and specificity of respectively 100% and 99%.
93. HPV circulating tumoral DNA quantification by droplet-based digital PCR: A promising predictive and prognostic biomarker for HPV-associated oropharyngeal cancers. Veyer D et al. December 2020.	OPC	66	ddPCR: Plasma samples were collected on 5 mL EDTA tubes. DNA was extracted using Qiaamp Minelute Virus Spin Kit (QIAGEN). ddPCR detection of HPV16 E6 gene was performed using RainDrop Digital PCR System (RainDance Technologies).	Forty-seven (71%) patients showed a positive pre-therapeutic HPV ctDNA at time of diagnosis. The quantity of HPV16 ctDNA at baseline, was significantly correlated with the T/N/M status or OPC stages. Moreover, all recurrences and the majority (83%) of death reported events occurred in patients with positive HPV16 ctDNA at baseline. Finally, the kinetic of pre-treatment/post-treatment HPV16 ctDNA was clearly associated with treatment success or failure.

## Abbreviations

HPVs	Human papillomaviruses
CEC	cervical cancer
OPC	oropharyngeal cancer
ct-DNA	Circulating tumor DNA
PCR	polymerase chain reaction
ddPCR	digital droplet-based PCR
HR	high-risk
ISH	in situ hybridization
TA-4	tumor associated antigen 4 (the squamous cell carcinoma antigen)
CEA	Carcino Embryonic Antigen
TPA	Tissue Polypeptide Antigen
TPS	Tissue Polypeptide Specific Antigen
CYFRA 21-1	cytokeratin-19 fragments
HNSCC	head and neck squamous cell cancer
cf-DNA	cell-free DNA
ct-DNA	cell-free tumoral DNA
NPC	nasopharyngeal carcinoma
HPV ct-DNA	circulating HPV DNA
HPV16 ct-DNA	circulating tumor HPV16 DNA
AUC	area under the curve
dPCR	digital PCR
NGS	next generation sequencing
TIL	tumor infiltrating lymphocyte therapy
EBV	Epstein-Barr Virus
ASCC	anal squamous cell carcinoma
CNVs	copy number variation

## Appendix A

The search terms were organized in the following formula: “(((((carcinoma of the cervix) OR (cervical cancer)) OR ((oropharyngeal cancer) OR (head and neck squamous cellular cancer))) AND (((early diagnosis) OR (treatment)) OR (surveillance))) AND (((PCR) OR (droplet digital PCR)) OR (next generation sequencing))) OR ((((human papillomavirus) OR (human papillomavirus DNA)) AND (cell free tumor DNA)) OR (circulating HPV DNA))”.

These research terms corresponded to the following MeSH terms: ((((“carcinoma”[MeSH Terms] OR “carcinoma”[All Fields] OR “carcinomas”[All Fields] OR “carcinoma s”[All Fields]) AND (“cervix uteri”[MeSH Terms] OR (“cervix”[All Fields] AND “uteri”[All Fields]) OR “cervix uteri”[All Fields] OR “cervix”[All Fields] OR “cervixes”[All Fields])) OR (“uterine cervical neoplasms”[MeSH Terms] OR (“uterine”[All Fields] AND “cervical”[All Fields] AND “neoplasms”[All Fields]) OR “uterine cervical neoplasms”[All Fields] OR (“cervical”[All Fields] AND “cancer”[All Fields]) OR “cervical cancer”[All Fields]) OR (“oropharyngeal neoplasms”[MeSH Terms] OR (“oropharyngeal”[All Fields] AND “neoplasms”[All Fields]) OR “oropharyngeal neoplasms”[All Fields] OR (“oropharyngeal”[All Fields] AND “cancer”[All Fields]) OR “oropharyngeal cancer”[All Fields] OR ((“head neck”[Journal] OR (“head”[All Fields] AND “and”[All Fields] AND “neck”[All Fields]) OR “head and neck”[All Fields]) AND “squamous”[All Fields] AND (“cells”[MeSH Terms] OR “cells”[All Fields] OR “cellular”[All Fields]) AND (“cancer s”[All Fields] OR “cancerated”[All Fields] OR “canceration”[All Fields] OR “cancerization”[All Fields] OR “cancerized”[All Fields] OR “cancerous”[All Fields] OR “neoplasms”[MeSH Terms] OR “neoplasms”[All Fields] OR “cancer”[All Fields] OR “cancers”[All Fields])))) AND (“early diagnosis”[MeSH Terms] OR (“early”[All Fields] AND “diagnosis”[All Fields]) OR “early diagnosis”[All Fields] OR (“therapeutics”[MeSH Terms] OR “therapeutics”[All Fields] OR “treatments”[All Fields] OR “therapy”[MeSH Subheading] OR “therapy”[All Fields] OR “treatment”[All Fields] OR “treatment s”[All Fields]) OR (“epidemiology”[MeSH Subheading] OR “epidemiology”[All Fields] OR “surveillance”[All Fields] OR “epidemiology”[MeSH Terms] OR “surveilance”[All Fields] OR “surveillances”[All Fields] OR “surveilled”[All Fields] OR “surveillence”[All Fields])) AND (“PCR”[All Fields] OR ((“droplet”[All Fields] OR “droplet s”[All Fields] OR “droplets”[All Fields]) AND (“digital”[All Fields] OR “digitalisation”[All Fields] OR “digitalised”[All Fields] OR “digitalization”[All Fields] OR “digitalize”[All Fields] OR “digitalized”[All Fields] OR “digitalizer”[All Fields] OR “digitalizing”[All Fields] OR “digitally”[All Fields] OR “digitals”[All Fields] OR “digitization”[All Fields] OR “digitizations”[All Fields] OR “digitize”[All Fields] OR “digitized”[All Fields] OR “digitizer”[All Fields] OR “digitizers”[All Fields] OR “digitizes”[All Fields] OR “digitizing”[All Fields]) AND “PCR”[All Fields]) OR (“high throughput nucleotide sequencing”[MeSH Terms] OR (“high throughput”[All Fields] AND “nucleotide”[All Fields] AND “sequencing”[All Fields]) OR “high throughput nucleotide sequencing”[All Fields] OR (“next”[All Fields] AND “generation”[All Fields] AND “sequencing”[All Fields]) OR “next generation sequencing”[All Fields]))) OR (((“alphapapillomavirus”[MeSH Terms] OR “alphapapillomavirus”[All Fields] OR (“human”[All Fields] AND “papillomavirus”[All Fields]) OR “human papillomavirus”[All Fields] OR ((“alphapapillomavirus”[MeSH Terms] OR “alphapapillomavirus”[All Fields] OR (“human”[All Fields] AND “papillomavirus”[All Fields]) OR “human papillomavirus”[All Fields]) AND (“dna”[MeSH Terms] OR “dna”[All Fields]))) AND (“circulating tumor dna”[MeSH Terms] OR (“circulating”[All Fields] AND “tumor”[All Fields] AND “dna”[All Fields]) OR “circulating tumor dna”[All Fields] OR (“cell”[All Fields] AND “free”[All Fields] AND “tumor”[All Fields] AND “dna”[All Fields]) OR “cell free tumor dna”[All Fields])) OR ((“blood circulation”[MeSH Terms] OR (“blood”[All Fields] AND “circulation”[All Fields]) OR “blood circulation”[All Fields] OR “circulation”[All Fields] OR “circulations”[All Fields] OR “circulate”[All Fields] OR “circulated”[All Fields] OR “circulates”[All Fields] OR “circulating”[All Fields]) AND “HPV”[All Fields] AND (“dna”[MeSH Terms] OR “dna”[All Fields]))).

Filters were used to restrict the selected studies to humans. No language restrictions were posed. Results were confined to the following article types: clinical study, clinical trial phase II; clinical trial phase III; clinical trial phase IV; guideline, meta-analysis, observational study, randomized controlled trial, systematic review and validation study. We also included in our research proceedings from national and international inherent meetings hold within the past 5 years. For the articles selected, reference lists were screened to identify additional relevant literature.
